# Isolation of MERS Coronavirus from a Dromedary Camel, Qatar, 2014

**DOI:** 10.3201/eid2008.140663

**Published:** 2014-08

**Authors:** V. Stalin Raj, Elmoubasher A.B.A. Farag, Chantal B.E.M. Reusken, Mart M. Lamers, Suzan D. Pas, Jolanda Voermans, Saskia L. Smits, Albert D.M.E. Osterhaus, Naema Al-Mawlawi, Hamad E. Al-Romaihi, Mohd M. AlHajri, Ahmed M. El-Sayed, Khaled A. Mohran, Hazem Ghobashy, Farhoud Alhajri, Mohamed Al-Thani, Salih A. Al-Marri, Mamdouh M. El-Maghraby, Marion P.G. Koopmans, Bart L. Haagmans

**Affiliations:** Erasmus Medical Center, Rotterdam, the Netherlands (V. Stalin Raj, C.B.E.M. Reusken, M.M. Lamers, S.D. Pas, J. Voermans, S.L. Smits, A.D.M.E. Osterhaus, M.P.G. Koopmans, B.L. Haagmans);; Supreme Council of Health, Doha , Qatar (E.A.B.A. Farag, H.E. Al-Romaihi, M.M. AlHajri, A.M. El-Sayed, M. Al-Thani, S.A. Al-Marri);; Hamad Medical Corporation, Doha (N. Al-Mawlawi);; Ministry of Environment, Doha (K.A. Mohran, H. Ghobashy, F. Alhajri, M.M. El-Maghraby)

**Keywords:** coronavirus, MERS, camel, viruses, Qatar

## Abstract

We obtained the full genome of Middle East respiratory syndrome coronavirus (MERS-CoV) from a camel in Qatar. This virus is highly similar to the human England/Qatar 1 virus isolated in 2012. The MERS-CoV from the camel efficiently replicated in human cells, providing further evidence for the zoonotic potential of MERS-CoV from camels.

Middle East respiratory syndrome coronavirus (MERS-CoV) is a novel coronavirus that can cause severe lower respiratory tract infection in humans (*1*,*2*). MERS-CoV clusters with viruses in the genus *Betacoronavirus*; the closest relative to this virus is bat CoVs clade 2c (*3*). Although bats are believed to carry different CoV ancestors, antibody reactivity against MERS-CoV has been found in serum from dromedary camels from countries within the Arabian Peninsula (*4*–*7*), Egypt (*8*), and the Canary Islands (*4*). More recently, MERS-CoVs that phylogenetically cluster with human MERS-CoVs were detected in camels from Qatar, Saudi Arabia, and Egypt (*7*,*9*–*12*). To further characterize MERS-CoV from camels, we screened nose swab samples from camels in Qatar.

## The Study

In February 2014, nasal swab samples were collected from 53 healthy dromedary camels in Doha, Qatar. After sampling, swabs were put into tubes containing viral transport medium and stored at −80°C until shipment to the Netherlands on dry ice, as described (*9*).

Total nucleic acids from nasal swabs were isolated by using the MagnaPure 96 total nucleic acid isolation kit (Roche, Mannheim, Germany), and samples were tested for MERS-CoV by using 2 TaqMan assays: 1 for the envelope (upE) and 1 for the nucleocapsid gene (N), as described previously (*9*,*13*). In each assay we detected MERS-CoV RNA in a sample from an 8-month-old camel. The cycle threshold of the positive sample was 12.9 in the upE assay and 11.3 in the N assay.

For further genomic characterization, RNA was isolated from 50 μL of 1 swab sample with the QIAamp Viral RNA Mini Kit (QIAGEN, Hilden, Germany), eluted in 60 μL water, and reverse transcribed with the Superscript III First-Strand Synthesis System (Life Technologies (Bleiswijk, the Netherlands) with random hexamers. The MERS-CoV genome was amplified by using MERS-CoV–specific overlapping primer sets as described previously (*3*). Amplified MERS-CoV fragments were sequenced directly on both strands by using the BigDye Terminator version 3.1 Cycle Sequencing kit on an ABI PRISM 3100 genetic analyzer (Applied Biosystems, Bleiswijk, the Netherlands). To obtain the 5′ and 3′ ends, we used the FirstChoice RLM-RACE kit (Ambion, Bleiswijk, the Netherlands) according to the manufacturer’s protocols. Using overlapping sequence fragments, we assembled the complete MERS-CoV genome, except for 1 nt potentially missing at the 5′ end.

The genome was 30,117 nt long, including 12 nt at the 3′ poly A tail (MERS-CoV camel/Qatar_2_2014, GenBank accession no. KJ650098). Similar to the genome of human MERS-CoV isolates, the genome of camel MERS-CoV isolates contains 10 complete open reading frames (ORFs) (ORF 1ab, spike, ORF3, ORF4a, ORF4b, ORF5, envelope, membrane, nucleocapsid, and ORF8b), 8 transcription-regulatory sequences, and 2 terminal untranslated regions. The alignment of the camel MERS-CoV with known human MERS-CoVs, including 1 near-complete camel MERS-CoV (NRCE_HKU205) sequence, showed overall nucleotide identities of 99.5%–99.9% between camel and human MERS-CoV isolates from different geographic regions.

**Figure 1:**
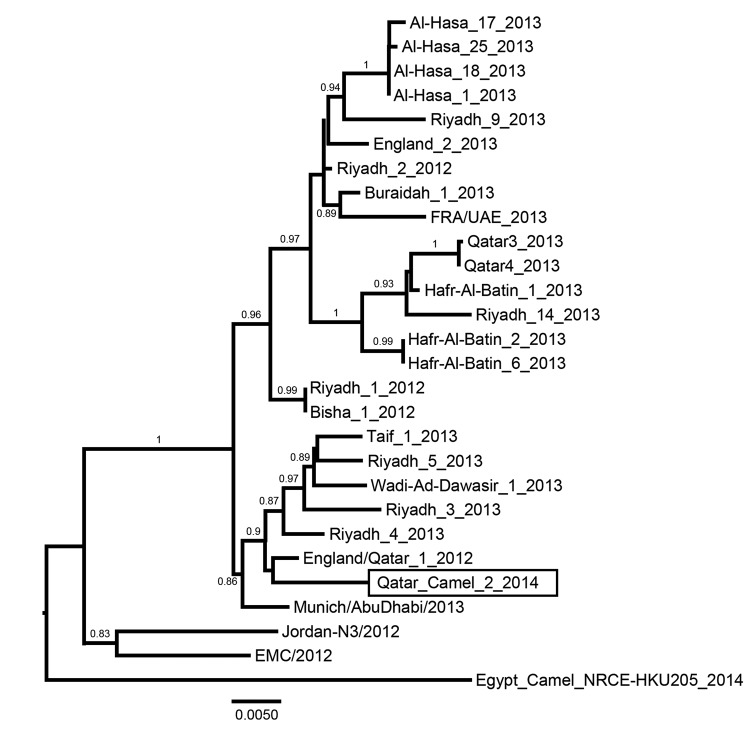
Phylogenetic analysis of Middle East respiratory syndrome coronaviruses (MERS-CoVs). Genome sequences of representative isolates were aligned by using ClustalW, and a phylogenetic tree was constructed by using the PhyML method in Seaview 4 (all 3 software packages can be found at http://pbil.univ-lyon1.fr/software/seaview) and was visualized in FigTree version 1.3.1 (http://tree.bio.ed.ac.uk/software/figtree/). Values at branches show the result of the approximate likelihood ratio; values <0.70 are not shown. The MERS-CoV isolated from a dromedary camel in Qatar in 2014 is depicted in a rectangle. Scale bar indicates nucleotide substitutions per site.

Phylogenetic analysis of the complete genome clearly showed that MERS-CoV camel/Qatar_2_2014 is highly similar to human MERS-CoV; the closest relative to camel MERS-CoV was England/Qatar1 2012 (99.9% identity) (Figure 1), and it was clearly distinct from the camel MERS-CoV (99.5% identity) isolated from camels at a different location in Qatar and in Egypt (*9*,*11*). Comparison of spike protein amino acid sequences from various human and camel isolates showed that this protein is highly conserved between this camel virus and other human isolates (online Technical Appendix Table, wwwnc.cdc.gov/EID/article/20/8/14-0663-Techapp1.pdf).

In addition, most amino acid residues critical for receptor binding (*14*) are identical in human and camel isolates, except for L506F in England/Qatar1. The biologic relevance of this mutation has not been investigated. The presence of arginine at position 1020 in the camel virus isolate might indicate that selective pressure at this site has probably not taken place as previously postulated. The fact that a MERS-CoV from a camel is highly similar to that from a human patient who probably became infected >1 year earlier in the same region suggests that this virus is maintained within camel populations and further supports the hypothesis that MERS-CoV can be transmitted from camels to humans.

To test for the presence of infectious virus, we titrated the swab sample on Vero cells (ATCC no. CCL-81). After 48 hours, we observed cytopathic changes in cells (320 50% tissue culture infectious dose/mL). After isolation, the passage-3 virus stock was used for all subsequent experiments.

To check for adaptive mutations obtained during cell culture, we used 454 deep-sequencing technology (Roche, Indianapolis, IN, USA) to analyze the full-genome sequence as described elsewhere (*3*). A total of 57,655 sequence reads were obtained, of which 17,056 were specific for MERS-CoV, revealing ≈99.77% of the virus genome. Genome coverage ranged from 1 to 2,082 reads at single nucleotide positions. Gaps or regions with coverage of <4 reads were confirmed by Sanger sequencing. When the genome of the passaged virus was aligned with the genome of the initial clinical isolate, we did not observe any mutations acquired during passaging.

**Figure 2:**
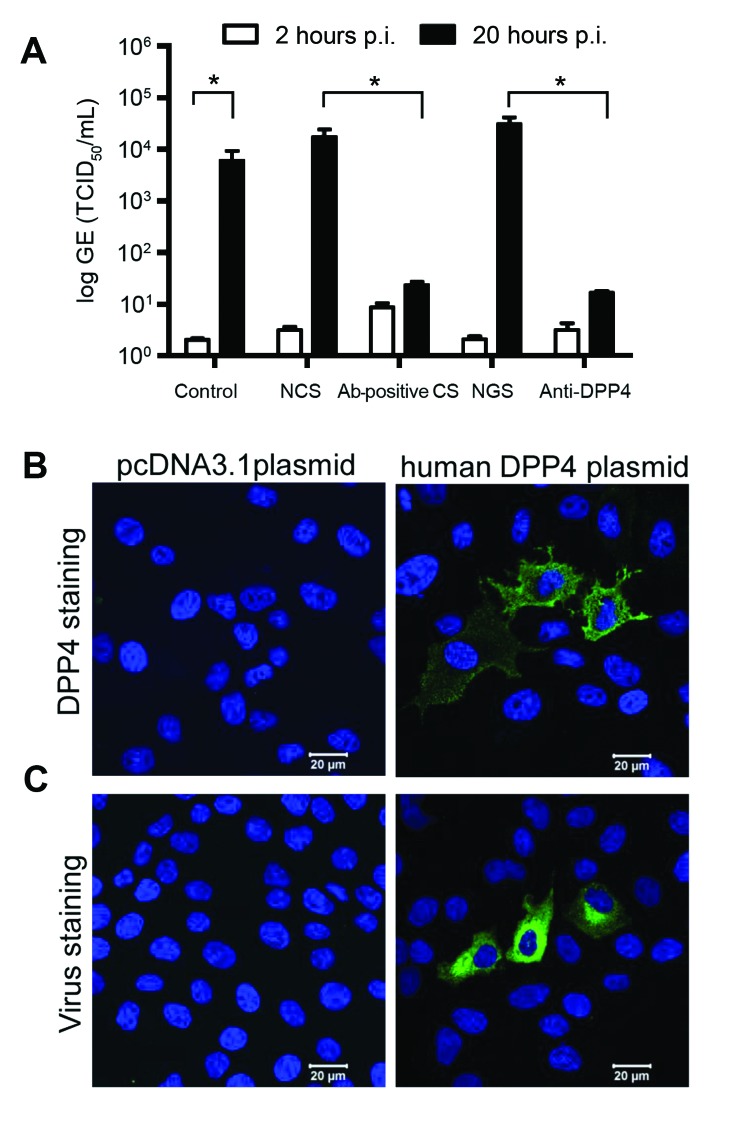
Middle East respiratory syndrome coronavirus (MERS-CoV) from camel replicates in human hepatoma (Huh-7) cells and uses human DPP4 as entry receptor. Huh-7 cells were inoculated with camel MERS-CoV and left for 1 h. Next, cells were washed twice, and supernatant was collected at 2 h (open bars) and 20 h (closed bars) before being tested for MERS-CoV RNA by using a TaqMan assay. We analyzed control camel MERS-CoV–infected cells, cells inoculated with camel MERS-CoV in the presence of normal camel serum (NCS), MERS-CoV–antibody positive camel serum (Ab-positive CS), normal goat serum (NGS), and anti-DPP4 polyclonal antibody–treated cells. Results are expressed as genome equivalents (GE), 50% tissue culture infective dose (TCID_50_/mL) (A). MDCK cells transfected with plasmid-encoding human DPP4 or a control plasmid (pcDNA) were stained with polyclonal antibody against human DPP4 (B) or inoculated with camel MERS-CoV and fixed 20 h after inoculation (p.i.) and stained for viral antigen (C).

To further functionally characterize this virus isolate, we subsequently inoculated human hepatoma (Huh-7) cells with MERS-CoV camel/Qatar_2_2014. After 2 days, virus-induced cytopathic effects were observed in the inoculated cell cultures (Technical Appendix Figure). In addition, a strong increase in virus titer was measured in the cell supernatant (Figure 2, panel A); produced virus could be passaged (not shown). Virus production in Huh-7 cells was blocked by preincubating camel MERS-CoV with a 1:200 dilution of serum from MERS-CoV antibody–positive camels (*9*) but not with seronegative camel serum (*4*) (Figure 2, panel A). Infection of Huh-7 cells could also be blocked by preincubation of cells with polyclonal antiserum against human DPP4 but not with control serum (Figure 2, panel A). Furthermore, transfection of nonsusceptible MDCK cells with human DPP4 (Figure 2, panel B), but not with empty vector, conferred susceptibility to infection with camel MERS-CoV (Figure 2, panel C). These data demonstrate that the MERS-CoV obtained from a dromedary camel is able to replicate in human cells and uses DPP4 as entry receptor, similar to MERS-CoV isolates obtained from human patients (*15*).

## Conclusions

We isolated MERS-CoV from the nasal cavity of 1 dromedary camel and demonstrated its infectiousness. Further studies are needed to test whether camels infected at a young age are more likely than adult dromedary camels to excrete infectious virus, possibly because of the MERS-CoV seronegative status of the younger camels. In addition, our results add to recent findings that MERS-CoVs from camels and humans are nearly identical (*9*–*11*). As might be expected from the high level of conservation in the critical interacting amino acids in the receptor-binding domain of the camel and human MERS-CoV isolates (online Technical Appendix Table), we show that camel MERS-CoV can infect human Huh-7 cells by using the same entry receptor as the human MERS-CoV isolates (*15*). Collectively, combined with the observation that the sequence of this virus was most closely related to that of a virus from a human patient who acquired MERS-CoV in Qatar a year earlier, these data support the hypothesis that dromedary camels are a reservoir for MERS-CoV and can transmit the infection to humans. However, whether exposure of humans to camels directly can lead to human infection cannot be concluded from our data. We are not aware of a connection between the camel population sampled in this study and the patient infected with MERS-CoV England/Qatar 1. Future epidemiologic studies are needed to investigate whether contact with camels or camel products constitutes a risk factor for MERS-CoV infection.

Technical Appendix Middle East respiratory syndrome coronavirus (MERS-CoV) in human hepatoma cells; variable amino acids in the MERS-CoV protein in different isolates.
