# Supplementary material for: Isolation of MERS Coronavirus from a Dromedary Camel, Qatar, 2014
Source: Emerg Infect Dis. 2014 Aug;20(8):1339–42. doi: 10.3201/eid2008.140663 (PMC4111206; doi:10.3201/eid2008.140663)
Supplement: Technical Appendix — Middle East respiratory syndrome coronavirus (MERS-CoV) in human hepatoma cells; variable amino acids in the MERS-CoV protein in different isolates. [file 14-0663-Techapp-s1.pdf]

Article DOI: http://dx.doi.org/10.3201/eid2008.140663

Isolation of MERS Coronavirus from a Dromedary Camel, Qatar, 2014

Technical Appendix

Appendix Table. Variable amino acids in the Middle East respiratory syndrome coronavirus spike protein in different isolates

	23	26	51	94	125	158	194	201	301	431	434	460	482	506	509	534	666	696	756	886	888	914	918	1020	1083	1158	1193	1208	1227	1333	1346
**EMC_2012**	P	V	S	G	N	D	H	Y	R	A	I	A	A	L	D	W	E	M	E	A	S	Q	A	Q	A	A	A	Q	T	C	E
**Qatar_Camel_2_2014**	P	V	S	G	N	D	H	H[a]	R	A	I	A	A	L	D	W	E	M	E	A	S	Q	A	R	A	A	A	Q	T	C	E
**Al-Hasa_1_2013**	P	V	S	G	N	D	H	Y	R	A	I	A	A	L	D	W	E	M	E	A	S	Q	A	R	A	A	A	H	T	C	E
**Al-Hasa_17_2013**	P	V	S	G	N	D	H	Y	R	A	I	A	A	L	D	W	E	M	E	A	S	Q	A	R	A	A	A	H	T	C	E
**Al-Hasa_18_2013**	P	V	S	G	N	D	H	Y	R	A	I	A	A	L	D	W	E	M	E	A	S	Q	A	R	A	A	A	H	T	C	E
**Al-Hasa_21_2013**	P	V	S	G	N	D	H	Y	R	A	I	A	A	L	D	W	E	M	E	A	S	Q	A	R	S	A	A	H	T	C	E
**Al-Hasa_25_2013**	P	V	S	G	N	D	H	Y	R	A	I	A	A	L	D	W	E	M	E	A	S	Q	A	R	A	A	A	H	T	C	E
**Bisha_1_2012**	P	V	S	G	N	D	H	Y	R	A	I	A	A	L	G	W	E	M	E	A	S	Q	A	R	A	A	A	Q	T	C	E
**Buraidah_1_2013**	P	V	S	G	N	D	H	Y	R	A	I	A	A	L	D	W	E	M	E	A	S	Q	A	R	A	A	A	Q	I	C	E
**Egypt_Camel_HKU205_2014**	L	A	S	G	N	D	Y	Y	R	A	S	A	A	L	D	W	K	T	Q	S	I	Q	S	Q	A	S	A	Q	T	R	E
**England/Qatar_1_2012**	P	V	S	G	N	D	H	Y	R	A	I	A	A	F[b]	D	W	E	M	E	A	S	Q	A	H	A	A	A	Q	T	C	E
**England_2_2013**	P	V	S	G	N	D	H	Y	R	A	I	A	A	L	D	W	E	M	E	A	S	Q	A	R	A	A	A	Q	T	C	E
**FRA/UAE_2013**	P	V	S	G	Y	D	H	Y	R	A	I	A	A	L	D	W	E	M	E	A	S	H	A	R	A	A	S	Q	T	C	E
**Hafr-Al-Batin_1_2013**	P	V	S	G	N	Y	H	Y	R	A	I	A	A	L	D	W	E	M	E	A	S	Q	A	R	A	A	A	Q	T	C	E
**Hafr-Al-Batin_2_2013**	P	V	S	G	N	Y	H	Y	R	A	I	A	A	L	D	W	E	M	E	A	S	Q	A	R	A	A	A	Q	T	C	E
**Hafr-Al-Batin_6_2013**	P	V	S	G	N	Y	H	Y	R	A	I	A	A	L	D	W	E	M	E	A	S	Q	A	R	A	A	A	Q	T	C	E
**Jordan-N3/2012**	P	V	S	V	N	D	Y	Y	L	A	I	A	A	L	D	W	E	M	E	A	S	Q	A	Q	A	S	A	Q	T	C	E
**Munich/AbuDhabi/2013**	P	I	S	G	N	D	H	Y	R	A	I	A	A	L	D	W	E	M	E	A	S	Q	A	H	A	A	A	Q	T	C	E
**Qatar3_2013**	P	V	S	G	N	D	H	Y	R	A	I	F	A	L	D	W	E	M	E	A	S	Q	A	R	A	A	A	Q	T	C	E
**Qatar4_2013**	P	V	S	G	N	D	H	Y	R	A	I	F	A	L	D	W	E	M	E	A	S	Q	A	R	A	A	A	Q	T	C	E
**Riyadh_1_2012**	P	V	S	G	N	D	H	Y	R	A	I	A	A	L	G	W	E	M	E	A	S	Q	A	R	A	A	A	Q	T	C	E
**Riyadh_14_2013**	P	V	S	G	N	D	H	Y	R	A	I	A	A	L	D	W	E	M	E	A	S	Q	A	R	A	A	A	Q	T	C	E
**Riyadh_2_2012**	P	V	S	G	N	D	H	Y	R	A	I	A	A	L	D	A	E	M	E	A	S	Q	A	R	A	A	A	Q	T	C	E
**Riyadh_3_2013**	P	V	S	G	N	D	H	Y	R	A	I	A	A	L	D	W	E	M	E	A	S	Q	A	H	A	A	A	Q	T	C	E
**Riyadh_4_2013**	P	V	S	G	N	D	H	Y	R	A	I	A	A	L	D	W	E	M	E	A	S	Q	A	H	A	A	A	Q	T	C	E
**Riyadh_5_2013**	P	V	F	G	N	D	H	Y	R	A	I	A	A	L	D	W	E	M	E	A	S	Q	A	H	A	A	A	Q	T	C	E
**Riyadh_9_2013**	P	V	S	G	N	D	H	Y	R	P	I	A	V	L	D	W	E	M	E	A	S	Q	A	R	A	A	A	Q	T	C	E
**Taif_1_2013**	P	V	S	G	N	D	H	Y	R	A	I	A	A	L	D	W	E	M	E	A	S	Q	A	H	A	A	A	Q	T	C	E
**Wadi-Ad-Dawasir_1_2013**	P	V	S	G	N	D	H	Y	R	A	I	A	A	L	D	W	E	M	E	A	S	Q	A	H	A	A	A	Q	T	C	Q
**Consensus**	P	V	S	G	N	D	H	Y	R	A	I	A	A	L	D	W	E	M	E	A	S	Q	A	R	A	A	A	Q	T	C	E

[a]Amino acid residues in the Middle East respiratory syndrome (MERS-CoV) spike protein that differ from the EMC/2012 isolate are displayed in red.
[b]Critical amino acid in binding to DPP4 receptor, displayed in green.

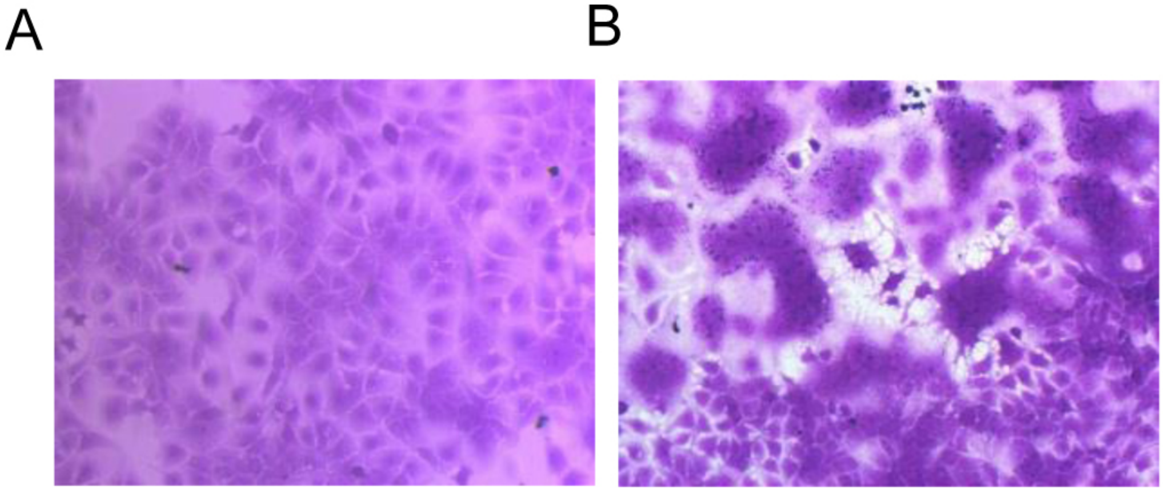

Appendix Figure. Middle East respiratory syndrome coronavirus (MERS-CoV) from camel replicates in human hepatoma (Huh-7) cells. Mock-inoculated cells (A) or cells inoculated with camel MERS-CoV (B) were fixed 40 hours after inoculation and stained with crystal violet.
